# Primary Tumor Vascularity, HIF-1α and VEGF expression in vulvar squamous cell carcinomas: their relationships with clinicopathological characteristics and prognostic impact

**DOI:** 10.1186/1471-2407-13-506

**Published:** 2013-10-29

**Authors:** Hari Prasad Dhakal, Jahn M Nesland, Mette Førsund, Claes G Trope, Ruth Holm

**Affiliations:** 1 Department of Pathology, The Norwegian Radium Hospital, Oslo University Hospital and Medical Faculty, University of Oslo, Oslo, Norway; 2 Department of Obstetrics and Gynecology, The Norwegian Radium Hospital, Oslo University Hospital and Medical Faculty, University of Oslo, Oslo, Norway

**Keywords:** Vulvar squamous cell carcinoma, HIF-1α, Immunohistochemistry, Tumor vascularity, Chalkley method

## Abstract

**Background:**

Increased vascularity is a crucial event in the tumor progression and has prognostic significance in various cancers. However, the ultimate role of angiogenesis in the pathogenesis and clinical outcome of vulvar carcinoma patients is still not settled.

**Methods:**

Tumor vascularity using CD34 stained slides measured by Chalkley counting method as well as hypoxia-inducible factor (HIF)-1α and vascular endothelial growth factor (VEGF) immunoexpression was examined in 158 vulvar squamous cell carcinomas. Associations between vascular Chalkley count, HIF-1α and VEGF expression and clinicopathological factors and clinical outcome were evaluated.

**Results:**

High CD34 Chalkley count was found to correlate with larger tumor diameter (*P* = 0.002), deep invasion (*P* < 0.001) and HIF-1α (*P* = 0.04), whereas high VEGF expression correlate significantly with poor tumor differentiation (*P* = 0.007). No significant association between CD34 Chalkley counts and VEGF expression and disease-specific survival was observed. High HIF-1α expression showed better disease specific survival in both univariate and multivariate analyses (*P* = 0.001).

**Conclusions:**

A significant association between high tumor vascularity and larger tumor size as well as deeper tumor invasion suggests an important role of angiogenesis in the growth and progression of vulvar carcinomas. HIF-1α expression in vulvar carcinomas was a statistically independent prognostic factor.

## Background

Vulvar carcinoma is accounting for 3-5% of all gynecological cancer and with an incidence ranging from 1 to 2 per 100 000 person-years worldwide [1,2]. The median age of these patients has been about 70 years. However, recently vulvar carcinomas are seen more frequently in younger patients [3,4]. The prognostic evaluation and treatment of vulvar carcinoma patients have been primarily guided by the lymph node status, the size of the tumor, depth of invasion, stage of the disease and grades [5-7]. Radical surgery is the most common treatment, but is often accompanied with physical and psychological adverse effects [5,8]. In an attempt to reduce severe complications, a change to individualized therapy has been reported [9]. Thus, identification of new markers which indicate the tumor behavior would be important to guide treatment decisions.

Angiogenesis is a crucial event for tumor growth and progression beyond a tumor size of 1–2 mm. Therefore, tumor neovasculature makes an important target for antiangiogenic therapy [10,11]. Increased tumor vascularity has been shown to have prognostic significance in various cancers including vulvar cancer [12-15]. The role of increased tumor vascularity in disease progression of various malignant gynecologic lesions, including malignant vulvar lesions, has been described [16,17]. Its importance in vulvar cancer has been emphasized by the increased vascularity in preinvasive lesions and invasive vulvar carcinomas [15-21]. Vulvar carcinoma patients with increased vascularity were reported to have poor prognosis in some studies [6,15,19], whereas other showed no significance [20].

Hypoxia-inducible factor (HIF)-1α, a transcription factor, is a key regulator of angiogenesis when a growing tumor experiences hypoxic stress and acts through various intracellular signalling pathways. Such activation results in the secretion of vascular endothelial growth factor (VEGF) and other factors related to tumor metabolism necessary for hypoxia compensation and tumor cell survival [22]. It is known to be expressed in various solid tumors including vulvar squamous cell carcinomas [23-30]. The relation between primary tumor vascularity and HIF-1α expression in head and neck and oesophageal squamous cell carcinoma has been reported [24,25], and the prognostic impact of HIF-1α expression in cancer is varied [26,27,29-32]. HIF-1α expression investigated recently in normal epithelium, intraepithelial neoplasia and invasive carcinoma of vulva did not show significant differences [28]. To our knowledge, no study of HIF-1α expression and its connection with prognosis in vulvar carcinoma patients has been reported. VEGF, a potent angiogenic molecule over-expressed in a hypoxic state, is crucial to induce tumor angiogenesis and acts through the receptors VEGFR1 and VEGFR2 [22,33]. It is expressed in various human cancers including vulvar malignancy [21,28,34,35]. A significant variation in expression of VEGF in nonneoplastic epithelium, preneoplastic lesions and invasive squamous cell carcinoma of vulva has been described [21,28,35]. Its expression in vulvar cancer and relationship with vascularity has been reported [19]. The prognostic impact of VEGF expression in invasive vulvar carcinoma is still not settled [19,36].

In the present study, we have evaluated a large series of primary vulvar squamous cell carcinomas for primary tumor vascularity and expression of HIF-1α and VEGF and elucidated their relationships with various clinicopathological parameters and clinical outcome.

## Methods

### Patient materials

A retrospective study was performed on a cohort of 158 patients with vulvar squamous cell carcinoma. All patients had undergone a resection at The Norwegian Radium Hospital between 1977 and 2006. The median age at diagnosis was 75 years (range, 41–92 years). In 108 (68%) of these cases radical surgery (a total vulvectomy plus a bilateral inguinal lymphadenectomy) had been performed, whereas the remaining 50 (32%) patients had non-radical surgery. Postoperative therapy had been administered to 44 patients including irradiation in 40 (25%) cases and irradiation/chemotherapy in four (3%) cases. Seventy-four (47%) of the patients died as a result of their vulvar cancer. All patients were followed up from the time of their confirmed diagnosis until death or 1. September, 2009. The median follow-up time for patients still alive was 108 months (range, 43 to 347 months). All tumors were staged based on the new International Federation of Gynecology and the Obstetrics (FIGO) classification from 2009 [37]. The Regional Committee for Medical Research Ethics South of Norway (S-06012), The Social and Health Directorate (04/2639 and 06/1478) and The Data Inspectorate (04/01043) approved the current study protocol. In this study we have used paraffin embedded tumor tissue from vulvar cancer patients diagnosed between 1977 and 2006. As many of these patients are dead or very old we did not have the opportunity to obtain patient consent. Permission to perform this study without patient consent was obtained from The Social and Health Directorate (04/2639).

Histological specimens were reviewed by the co-author J.M.N. without access to any clinical information on the patients. The tumors were classified according to the World Health Organization recommendations [38]. All 158 tumors were classified as keratinizing/non-keratinizing squamous cell carcinomas.

### Immunohistochemistry

Three micrometer sections were processed for immunohistochemistry using the Dako EnVision™ Flex+ System (K8012; Dako, Glostrup, Denmark) and the Dako Autostainer. Deparaffinization and the unmasking of epitopes were performed using PT-Link (Dako) and EnVision™ Flex target retrieval solution at a high pH. After treatment with 0.03% hydrogen peroxide (H_2_O_2_) for 5 min to block endogenous peroxidase activity, the sections were incubated with monoclonal antibodies raised against CD34 (30 min at room temperature, clone QBEND-10, 1:1000, 1μg IgG_1_/ml) purchased from Monosan (Uden, The Netherlands), HIF-1α (over night at 4°C, clone 54/HIF-1α, 1:100, 2.5 μg IgG_1_/ml) purchased from BD Transduction Laboratories™ (San Jose, CA, USA) and VEGF (over night at 4°C, clone VG1, 1:100, 0.45 μg IgG_1_/ml) purchased from Dako. Then the slides were incubated with EnVision™ Flex+ mouse linker (15 min), EnVision™ Flex/HRP enzyme (30 min) and 3’3-diaminobenzidine tetrahydrochloride (DAB) (10 min). After counterstaining with hematoxylin the samples were dehydrated and mounted in Richard-Allan Scientific Cyto seal XYL (Thermo Scientific, Waltham, MA, USA). All of the sample series included positive controls known to be positive for CD34, HIF-1α and VEGF. As negative controls, the primary antibodies were replaced with mouse myeloma protein IgG_1_ at the equivalent concentration.

### Quantification of tumor vascularity

Chalkley method was used for quantification of tumor vascularity as recommended in a consensus meeting [39]. The method has been described in detail earlier [14]. Three most vascularized areas in the CD34 stained tumor section known as “hotspots” were identified under the low power magnification after scanning first at ×40 and then ×100 magnification following the Weidner’s method of selection of vascular hotspots [40]. Then a 25 point Chalkley eyepiece graticule fixed in one of the eyepieces of the microscope was applied to each vascular hotspot at ×200 magnification [Chalkley grid area of 0.1886 mm^2^ (Nikon microscope, Eclipse E400)] in such a way that maximum number of black dots in Chalkley graticule fell on or within immunostained microvessels. The number of these dots that have fallen on or within the immunostained microvessels were counted in each selected hotspot area and recorded as Chalkley count. Sclerotic and necrotic area was avoided and count was done in only invasive carcinoma including margin. The highest count among the 3 hotspots counts from each tumor was used for further analyses. Measurement of vascularity was performed without the knowledge of clinicopathological data or clinical outcome.

### Evaluation for HIF-1α and VEGF expression

Expression of HIF-1α was evaluated on immunostained slides semiquantitatively into four classes and only nuclear immunoreactivity of the tumor cells was taken into account. Due to similar staining intensity of the HIF-1α positive cases we did not consider the intensity of immunostaining. Based on the number of HIF-1α positively stained tumor cells, tumors were grouped into: 0% of the cells; < 10% of the cells; 10-50% of the cells and > 50% of the cells. For further analyses, HIF-1α expression in nucleus in more than 50% of the tumor cells was considered as high. VEGF positive cases showed different staining intensity and both intensity and number of positive tumor cells were evaluated. Cytoplasmic expression of VEGF was categorized semiquantitatively on the basis of intensity of the signal (absent, 0; weak, 1; moderate, 2; strong, 3) and the percentage of positive tumor cells (absent, 0; < 10%, 1; 10-50%, 2; > 50%, 3). The composite score was calculated as fraction of positive tumor cells score multiplied by intensity score, and range from 0 to 9. For further analyses, cytoplasmic VEGF immunostaining with a composite score ≥ 6 was classified as high expression. Examination of immunostaining was performed in a blinded fashion with no knowledge of the clinicopathological variables and patient outcomes.

### Statistical analyses

The associations between the HIF-1α and VEGF expression and CD34 Chalkley counts of primary tumor vascularity and the clinicopathological variables were evaluated by the Pearson chi-square (χ^2^), Fisher’s exact test and linear-by-linear association as required. The disease-specific survival analysis, based on death from vulvar cancer only, was performed using the Kaplan Meier method and *P* value computed by log-rank test. A Cox proportional hazards regression model was used for both univariate and multivariate evaluation of survival rates. In the multivariate analysis, a backward regression was performed and variables with a *P* ≤ 0.05 in univariate survival analysis were included in the model. The vulvar carcinoma tissues in our cohort have been collected over an extensive period from 1977–2006. Due to the large variation in storage time and given that the fixation protocol for these tissues up to 1987 was acid formalin, whereas from 1987–2006 was buffered formalin, Mann–Whitney U test was performed to evaluate whether this has any influence on the CD34, HIF-1α and VEGF immunostaining. The Mann–Whitney U test showed that the distribution of CD34, HIF-1α and VEGF expression was the same between samples processed before and after 1987. All analyses were processed using the SPSS 18.0 statistical software package (SPSS, Chicago, IL). Statistical significance was considered for *P <* 0.05.

## Results

**Figure 1:**
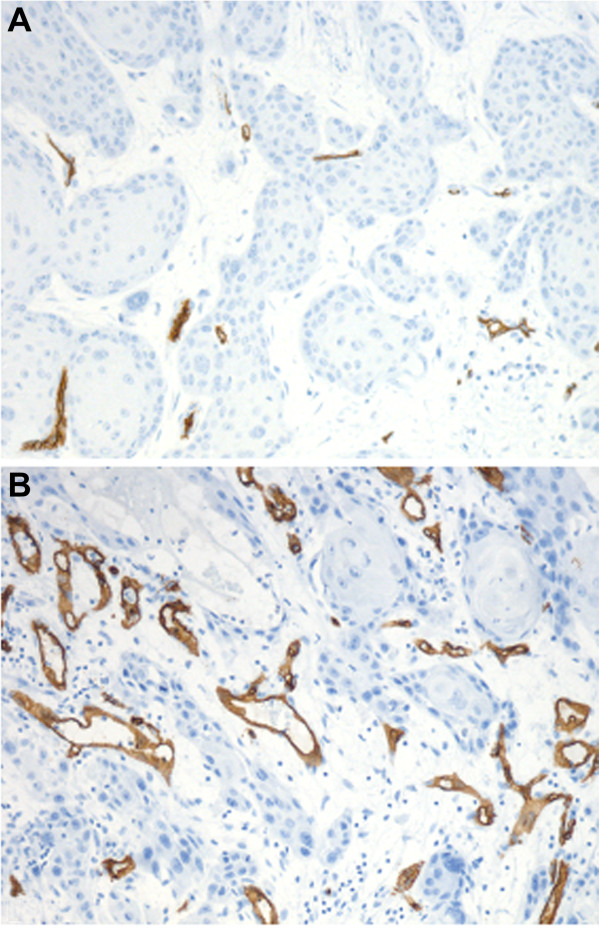
**Representative images of CD34 staining of primary vulvar carcinoma vascularization. (A)** Low vascularity (low Chalkley count) and **(B)** High vascularity (high Chalkley count). Images were taken by a Leica DFC 320 digital camera with a Plan-neofluar 10× objective lens in Axiophot microscope (Zeiss Germany).

**Figure 2:**
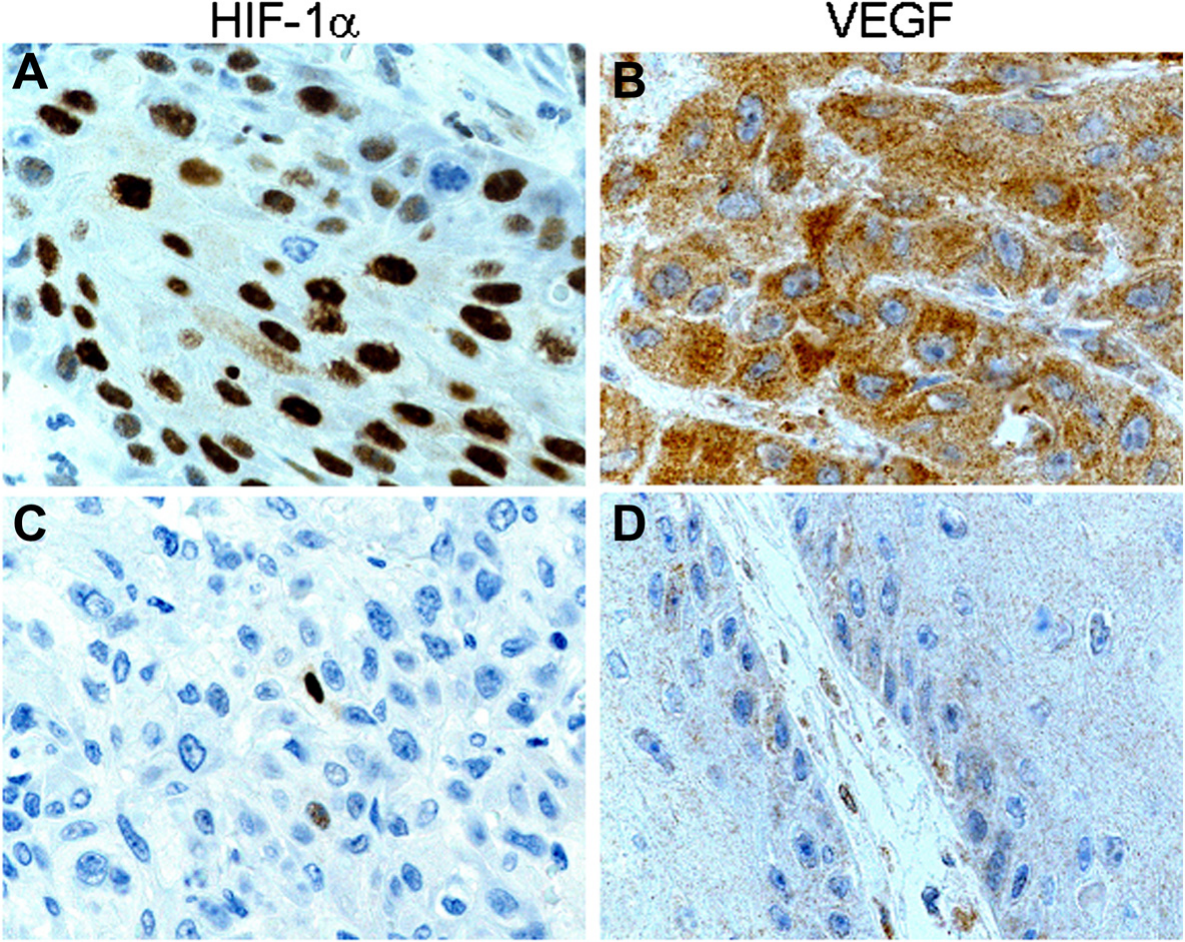
**Representative images of HIF-1α and VEGF immunoexpression in primary vulvar carcinoma. (A)** high HIF-α nuclear expression and **(B)** low HIF-α nuclear expression **(C)** high VEGF cytoplasmic staining and **(D)** low VEGF cytoplasmic staining. 40× objective lens.

Vascularization in vulvar squamous cell carcinoma was heterogenously distributed. Microvessels were located in the tumor stroma lying between the islands of tumor cells and the size and shape of the vessels greatly varied. The CD34 Chalkley counts for the vulvar carcinoma vascularity ranged from 3–14 (mean, 7.92; median, 8; SD, 2.29). Predefined cutoff value of 8 (median value) was used to dichotomize the tumor into high and low vascular groups. Low (Chalkley counts < 8) and high (Chalkley counts ≥ 8) vascularity was identified in 67 (42%) and 91 (58%) of the vulvar carcinomas, respectively (Figure 1A and B). In vulvar carcinomas, high HIF-1α immunostaining (> 50% tumor cells) in the nucleus was observed in 57 (36%) and low levels (≤ 50% tumor cells) in 101 (64%) cases (Figure 2A and B), whereas high VEGF expression (score ≥ 6) in the cytoplasm was identified in 63 (40%) and low low level (score < 6) in 95 (60%) cases (Figure 2C and D).

CD34 Chalkley count, HIF-1α and VEGF expression in relation to clinicopathological parameters are shown in Table 1. High CD34 Chalkley count was found to correlate significantly with larger tumor diameter (*P* = 0.002) and deeper invasion (*P* < 0.001), whereas high VEGF expression correlate significantly with poor tumor differentiation (*P* = 0.007). High level of HIF-1α was significantly correlated to high CD34 Chalkley counts (*P* = 0.04). VEGF expression did not show any association with CD34 Chalkley count and HIF-1α levels.

**Table 1:** CD34 Chalkley count, HIF-1α and VEGF expression in relation to clinicopathological variables in vulvar carcinomas

**Variable**	**Total**	**CD34 Chalkley count**			**HIF-1α**			**VEGF**		
	**N**	**Low**	**High (%)**	***P *** **value**	**Low**	**High (%)**	***P *** **value**	**Low**	**High (%)**	***P *** **value**
Age				0.25^1^			0.28^1^			0.68^1^
25–69	59	30	29 (49)		34	25 (42)		36	23 (39)	
70–84	81	29	52 (64)		55	26 (32)		46	35 (43)	
85+	18	8	10 (56)		12	6 (33)		13	5 (28)	
FIGO				0.67^2^			0.22^2^			0.08^2^
Ia	0	0	0 (0)		0	0 (0)		0	0 (0)	
Ib	77	35	42 (55)		51	26 (34)		48	29 (38)	
II	7	2	5 (71)		4	3 (43)		7	0 (0)	
IIIa	30	14	16 (53)		13	17 (57)		20	10 (33)	
IIIb	26	8	18 (69)		18	8 (31)		11	15 (58)	
IIIc	7	2	5 (71)		5	2 (29)		4	3 (43)	
IVa	1	1	0 (0)		1	0 (0)		0	1 (100)	
IVb	7	3	4 (57)		6	1 (14)		4	3 (43)	
Not available	3									
Lymph node metastasis				0.21^3^			0.54^3^			0.11^3^
None	87	39	48 (55)		58	29 (33)		55	31 (37)	
Unilateral	44	19	25 (57)		25	19 (43)		29	15 (34)	
Bilateral	24	6	18 (75)		15	9 (38)		10	14 (58)	
Not available	3									
Tumor diameter (cm)				0.002^1^			0.95^1^			0.98^1^
0.3–2.5	32	19	13 (41)		19	13 (41)		20	12 (38)	
2.6–4.0	51	24	27 (53)		33	16 (31)		30	21 (41)	
4.1–20.0	72	21	51 (71)		44	28 (39)		44	28 (39)	
Not available	3									
Tumor differentiation				0.07^3^			0.23^3^			0.007^3^
Well	35	19	16 (46)		26	9 (26)		19	16 (46)	
Moderate	92	32	60 (65)		54	38 (41)		64	28 (30)	
Poor	31	16	15 (48)		21	10 (32)		12	19 (61)	
Depth of invasion (mm)				<0.001^1^			0.58^1^			0.78^1^
0.0–4.0	26	19	7 (27)		17	9 (35)		15	11 (42)	
4.1–8.0	56	26	30 (54)		37	19 (34)		37	19 (34)	
8.1–40.0	74	20	54 (73)		45	29 (39)		43	31 (42)	
Not available	2									
Infiltration of vessel				0.55^3^			0.06^3^			0.73^3^
No	116	51	65 (56)		70	46 (40)		72	44 (38)	
Yes	39	15	24 (62)		30	9 (23)		23	16 (41)	
Not available	3									

^1^Linear-by-linear association.

^2^Fisher’s exact test.

^3^Pearson chi-square.

Low: CD34 Chalkley count < 8; High: CD34 Chalkley count ≥ 8.

Low: HIF-1α in ≤ 50% tumor cells; High: HIF-1α in > 50% tumor cells.

Low: VEGF score < 6, High: VEGF score ≥ 6.

**Figure 3:**
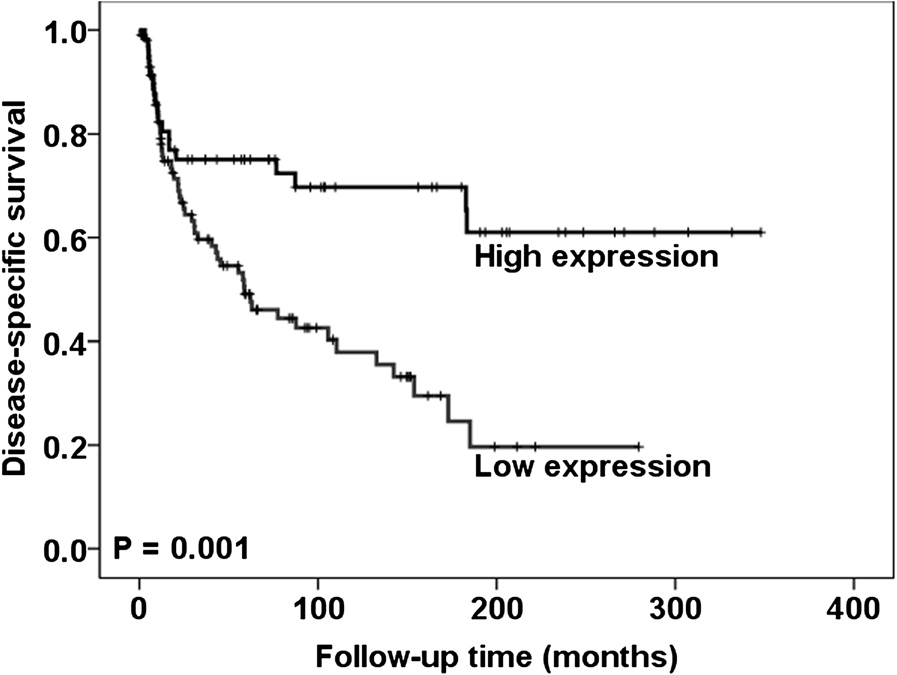
**Survival curves using the Kaplan-Meier method.** The Kaplan-Meier curve of disease-specific survival in relation to the HIF-1α showed that patients whose tumors expressed low levels of HIF-1α had a worse prognosis than those with high levels.

In univariate survival analysis, high HIF-1α expression was associated with better disease-specific survival (*P* = 0.001) (Figure 3), whereas no significant association between CD34 Chalkley counts and VEGF expression and disease-specific survival (*P* = 0.16 and *P* = 0.45, respectively) was observed. In multivariate analysis, lymph node metastases, age and HIF-1α expression retained independent prognostic significance (Table 2).

**Table 2:** Relative risk (RR) of dying from vulvar cancer

**Variables**	**Univariate analysis**			**Multivariate analysis**		
	**RR**	**95% CI**^**a**^	***p***	**RR**	**95% CI**^**a**^	***p***
Lymph node metastasis	1.99	1.49–2.65	<0.001	2.28	1.69–3.07	<0.001
Infiltration of vessel	2.20	1.36–3.58	0.001	-	-	-
Age	1.70	1.20–2.41	0.003	1.92	1.31–2.81	0.001
Tumor diameter	1.40	1.03–1.91	0.03	-	-	-
HIF-1α^b^	2.49	1.45–4.28	0.001	2.53	1.46–4.37	0.001

^a^95% confidence interval.

^b^Low: ≤ 50% tumor cells and high: > 50% tumor cells.

## Discussion

We observed that primary tumor vascularity, quantified by Chalkley method, had a significant association with tumor size and depth of invasion in invasive vulvar carcinomas. Tumor size has been reported to predict local lymph node metastasis [41] and is an important prognostic marker in vulvar cancer patients. Tumor size is at present used to stratify patients into different risk groups and acts as a determinant for surgical treatment [6,7]. In vulvar carcinomas, depth of tumor invasion is also indicative of the aggressiveness of primary tumor and is reported to be associated with lymph node metastases [41] and reduced survival [6]. Inguinofemoral lymph node status is the most powerful indicator of poor prognosis in vulvar cancer [42-44] and a significantly reduced survival in the current study has been confirmed. In the present study, no prognostic significance of tumor vascularity was observed for patients with vulvar carcinoma. This is in accordance with an earlier study on vulvar cancer [20], but in contrast to others [6,15,19]. These conflicting reports on primary tumor vascularity and prognosis might be due to methodological differences, different study cohort or biological factors [6,19,20]. We used the Chalkley counting method for vascular quantification which measures the relative vascular area [45], as recommended in a consensus meeting for quantification of vascularity in solid tumors [39], whereas in other studies microvessels have been counted manually [15,19,20] or using image analyses [6]. Moreover, other studies [6,15,19,20] had analysed smaller number of cases compared to our large series of vulvar carcinomas. Thus, our results of high tumor vascularity associated with larger tumor size and deeper invasion (known pathological markers for tumor aggressiveness) indicates angiogenesis as a marker for the aggressive behaviour of vulvar carcinoma.

HIF-1α, is a crucial molecule in inducing angiogenesis in growing tumor under hypoxic stress [22] and several reports have been published on relation between HIF-1α expression and angiogenesis in head and neck and oesophageal squamous cell carcinoma [24,25]. In present study, we did observe a positive association between HIF-1α expression and CD34 Chalkey count of primary tumor vascularity similar to a report for head and neck squamous cell carcinoma patients [25]. This confirms the role of HIF-1α for the initiation and the promotion of angiogenesis in vulvar cancer. High tumoral HIF-1α expression is reported to be associated with reduced survival in oral, oropharyngeal and cervical cancers [29,32,46]. In contrast, in the present study, a significantly improved survival of vulvar carcinoma patients with high HIF-1α expression was observed as reported for the squamous cell caricnoma in head and neck region, oral cavity and uterine cervix [26,27,30,31]. Other did not find prognostic significance in oesophageal squamous cell carcinoma [47]. Various factors are thought to affect the impact of HIF-1α activation in tumor behaviour [48] including methodology, cut off(s) and treatment modalities [26,27,30-32,47]. Lack of CAIX and Glut-I expression along with high HIF-1α expression in squamous cell carcinoma indicates alternative mechanism for HIF-1α upregulation [26]. Furthermore, we have shown that the patients in good prognosis group had >50% HIF-1α positive tumor cells as reported for its strong expression in squamous cell carcinoma of oral cavity [27]. Diffuse HIF-1α expression based on tumor types and its nonhypoxic activation through various genetic alterations that might result in different outcomes [22,26] may also explain our observation. Vascularization was heterogenously distributed in the tumor including tumor fronts and in the stromal tissue between the islands of tumor cells. Perinecrotic tumor cells distant from the supplying vessels under hypoxic stress express HIF-1α, whereas nonnecrotic tumor shows diffuse expression throughout the tumor including the tumor cells close to the blood vessels [29]. Despite, the heterogenous distribution of vascularity, our observation of positive association between HIF-1α and tumor vascularity suggests the HIF-1α induced angiogenesis. HIF-1α is known to induce expression of various genes including genes linked to cell survival, apoptosis, cellular proliferation [22]. Perhaps, the better outcome in patients with high HIF-1α expressing tumors might be due to its inhibitory role on tumor cells through induction of proapoptotic pathway [49,50].

Hypoxia markers; HIF-1α, GLUT-1, CA IX and VEGF are expressed in both vulvar preneoplastic lesions and invasive squamous cell carcinoma [28]. An increasing expression of VEGF from normal epithelium to premalignant lesion to invasive squamous cell carcinoma was found in vulva [28]. In the present study, high VEGF expression was significantly associated with only poor tumor differentiation, however, an other study reported no such association in vulvar carcinomas [19]. We did not find that the VEGF levels demonstrated prognostic significance, a result being different from a report by Obermair and colleagues [19]. A lack of correlation of VEGF with vascularity observed in the present series which is different from an earlier report [19], might be due to its possible non-angiogenic effects and/or autocrine role on tumor cells [51,52]. Alternative mechanisms of VEGF independent neoangiogenesis by inducing other potent angiogenic molecules like basic fibroblast growth factor and size related proainherent angiogenic effect on the tumor [53] may have resulted in nonsignificant relationship. We noted no association between VEGF and HIF-1α expression possibly due to an alternative non HIF-1α mechanism of VEGF induction [54]. The proangiogenic effect of VEGF is closely related to the tumor size and no impact on angiogenesis is found when tumor reaches to certain size [53].

There are several pitfalls associated to immunohistochemical methods. In addition, the handling of tissue specimens, such as fixation and storage time, may influence the immunohistochemical results [55]. The lack of consideration for these limitations may reduce the usefulness of immunohistochemical studies. In the present study, the fixation and storage time of the tissues did not influence the CD34, HIF-1α and VEGF immunostaining. Both false positive and negative results may limit the outcome of immunohistochemical studies. To reduce the possibility of false negative results we have used the EnVision™ Flex+ detection system reported to have a high sensitivity [56]. Furthermore, we have included positive controls in each run to exclude the possibility of false negative result due to methodological problems. To avoid nonspecific staining we extensively optimalized the dilutions of the primary antibodies used. In addition, negative controls, replacing the primary antibodies with the mouse myeloma protein IgG_1_, were included to exclude the possibility of false positive results. Despite the effort to quality secure immunostaining processes there are major limitations connected to immunohistochemistry between the studies that are linked to methodological differences including immunostaining procedures and scoring systems [57]. In the future it is clearly needed a standarization of immunohistochemical methodology and scoring systems.

## Conclusions

Our results show that high tumor vascularity in vulvar carcinoma is associated with larger tumor size and deeper invasion, indicating that it is a feature of aggressive tumor phenotype. High HIF-1α expression has favorable prognostic impact in vulvar carcinoma patients.

## Competing interests

Authors declare that they have no competing interests.

## Author’ contributions

HPD participated in the design of the study, quantified tumor vascularity and draft the manuscript. JMN participated in the design of the study, performed systematic pathologic review of vulvar carcinomas and revised the manuscript critically. MF carried out the immunohistochemistry and revised the manuscript critically. CGT collected clinical data, participated in interpretation of data and revised the manuscript critically. RH participated in the design of the study, protein, statistical and data analysis and helped to draft the manuscript. All authors read and approved the final manuscript.

## Pre-publication history

The pre-publication history for this paper can be accessed here:

http://www.biomedcentral.com/1471-2407/13/506/prepub
